# NOTCH-ing up nucleotide metabolism in T-cell acute lymphoblastic leukemia

**DOI:** 10.1038/s42003-021-02330-8

**Published:** 2021-06-29

**Authors:** Andrew Volk

**Affiliations:** 1 Department of Experimental Hematology and Cancer Biology, Cincinnati Children’s Hospital Medical Center, Cincinnati, OH USA; 2 Department of Pediatrics, University of Cincinnati College of Medicine, Cincinnati, OH USA

**Keywords:** Cancer metabolism, Acute lymphocytic leukaemia

## Abstract

In a recent issue of Science Advances, Srivastava et al. report a novel nucleotide biosynthesis regulatory role for UBR7 in NOTCH1-driven T-ALL. Here we will discuss their key findings and the implications for using cancer metabolism to understand the development and progression of T-ALL.

In the 1920s, the German physiologist Otto Warburg made the fundamental observation that most cancer cells do not rely on oxidative phosphorylation for energy production. Instead, and regardless the presence of oxygen, these tumors divert glucose for lactate production as a means to generate energy^1,2^. Now known as the “Warburg Effect”, it is one of the defining characteristics of cancer metabolism and has shaped the trajectory of cancer research for a century. Modern studies have built on this observation, and we have collectively amassed a catalogue of mutations in tumor suppressors and oncogenes that all have profound effects on cellular and tumor metabolism. In fact, we now consider metabolic reprogramming as a hallmark of cancer^3^. In this recent study by Srivastava et al., the authors follow in this tradition and suggest a novel therapeutic strategy for T-cell acute lymphoblastic leukemia (T-ALL) focused on disrupting the nucleotide synthesis pathway by targeting the ubiquitin protein ligase E3 component N-recognin 7 (UBR7)^4^.

T-ALL is an aggressive hematological malignancy that commonly affects pediatric patients and is characterized by rapid expansion of malignant T lymphocytes^5^. Although recent advances in therapy have increased the likelihood of surviving this disease to 80% or greater (90% in some studies), almost 20% of patients still succumb to this disease within 5 years^6,7^. NOTCH1 activating mutations are the most common drivers of T-ALL, accounting for nearly 60% of diagnosed cases^8^. Normal activation of NOTCH1 is achieved through proteolytic cleavage by gamma-secretase following NOTCH1 extracellular domain binding to its ligand. This releases the NOTCH1-intracellular domain (NICD) allowing it to translocate to the nucleus and activate target genes^9^. Oncogenic NOTCH1, as seen in T-ALL, is characterized by a wide variety of mutations that (1) uncouple the need for ligand binding to regulate NICD release and/or (2) stabilize the NICD protein-protein or protein-DNA interactions^9^. Gamma-secretase is the enzyme responsible for activating NICD through proteolytic cleavage, a necessary activity for NICD release in both normal and oncogenic NOTCH1. This observation has led to the concept of gamma-secretase inhibitors (GSI) as attractive therapeutic strategies for T-ALL. However, both the substantial toxicity of GSIs on other tissues and the relative ease of T-ALL tumors to mutate and develop resistance have highlighted the need for better therapeutic strategies to mitigate oncogenic NOTCH1 and treat T-ALL^10,11^.

Srivastava et al. identified the E3 ubiquitin ligase UBR7 as a NOTCH1 transcriptional target in the T-ALL cell line CUTLL1^4^. Analysis of the publicly available cancer cell line encyclopedia (CCLE) showed that UBR7 is maximally expressed in T-ALL as compared to other cancer types^4,12^. They further confirmed UBR7 overexpression in a wide-range of NOTCH1-driven T-ALL cell lines and patient samples. Consistent with NICD role as a transcription factor, the authors confirmed that NOTCH1 promotes UBR7 expression through NICD binding to the UBR7 promoter. Blocking NOTCH1 through pharmacological inhibition with a GSI led to a decrease in NOTCH1 binding to the UBR7 promoter and reduction in UBR7 expression. Direct experimental repression of UBR7 using shRNA in T-ALL models showed reductions in cell proliferation in vitro and reduced tumorigenesis in mouse models in vivo.

The most salient finding in this study is that UBR7 promotes T-ALL by regulating specific nucleotide biosynthesis pathways. Nucleotide biosynthesis is a fundamental and essential metabolic process that promotes cell proliferation and survival by ensuring the adequate raw material availability for nucleic acid synthesis. Cancer cells must meet the nucleotide demand associated with rapid cell proliferation^13^. Using an unbiased affinity purification-mass spectrometry based proteomic screen, the authors identified Phosphoribosyl Pyrophosphate (PRPS) 1 and 2 enzymes as robust interacting partners of UBR7^4^. The PRPS enzymes catalyze the first (and rate-limiting) step in the nucleotide biosynthesis pathway, making them essential regulators of nucleotide metabolism^14^. PRPS enzymes are normally found in complex with regulatory subunits, known as PRPS-associated protein (PRPSAP) 1 and 2^14^. The PRPSAPs have been thought to have an inhibitory effect on PRPS catalytic activity and the study demonstrates that UBR7 stabilizes the PRPS enzymes by promoting polyubiquitination-directed degradation of PRPSAP^4^. The study also shows that deficiency of UBR7 leads to a marked reduction in both PRPS1/2 levels^4^. Consistent with the decrease in PRPS enzyme levels, the levels of phosphoribosyl pyrophosphate (PRPP), the metabolic product of PRPS catalytic reaction, were also reduced. As a result, depletion of UBR7 also leads to depleted nucleotide pools and impaired nucleotide biosynthesis in T-ALL cell lines^4^. These findings are further substantiated by rescue experiments demonstrating the ability of ectopic *UBR7* to restore the proliferation and nucleotide biosynthesis defects caused by the depletion of endogenous UBR7^4^. The slow proliferation rates of T-ALL cell lines in absence of UBR7 are also rescued by ribose nucleoside supplementation, functionally bypassing the requirement of PRPS enzymes for nucleotide production, and strengthening the idea that UBR7 governs T-ALL cell proliferation through the regulation of nucleotide synthesis^4^.

While aberrant metabolism of tumors feed cell growth and proliferation, it also introduces a metabolic vulnerability that can be exploited for therapy^15^. Several metabolic pathways including glycolysis, pentose phosphate pathway, amino acid and nucleotide syntheses have been effectively targeted in leukemias^16,17^. Thiopurines including 6-mercaptopurine and 6-thioguanine are key drugs for ALL treatment, however certain mutations in *PRPS1* confer resistance to these drugs^18^. While these mutations render PRPS1 hyperactive, Srivastava et al suggest that since hyperactive PRPS1 is stabilized by UBR7, targeting UBR7-PRPS1 interaction may alleviate the thiopurine resistance in leukemia^4^.

As the field of cancer metabolism continues to evolve, the study from Srivastava et al. significantly advances our understanding of nucleotide metabolism in the context of NOTCH1-driven T-ALL. Previous studies have shown that AMPK can regulate PRPS-mediated nucleotide synthesis in glioblastoma and fibroblast lines^19^, but the molecular mechanisms regulating the function of these enzymes in the T-ALL setting have remained undefined. This work by Srivastava et al. demonstrates targeting NOTCH1 downstream effectors could augment GSI treatments and potentially avoid some of the inherent toxicities and resistances associated with this as a T-ALL therapy. In line with this idea, several studies highlighting NF-κB, mTOR, and glucocorticoid receptor inhibitors have been shown to synergize with NOTCH1 inhibition with significantly less gastrointestinal toxicity^11^. The fine tuning of PRPS and PRPSAP by UBR7 further ensures a functional nucleotide biosynthesis pathway that supports T-ALL progression (Fig. 1)^4^. The evidence from this study showing silencing of UBR7 mitigates tumor formation from both GSI sensitive and resistant T-ALL cell lines, opens a new therapeutic window and suggest future efforts should be directed towards developing clinical inhibitors against UBR7. These observations presented in this study could lead to exciting prospects for potential future therapies and hold great promise to offer valuable insights into metabolic regulation of cancers.

**Fig. 1:**
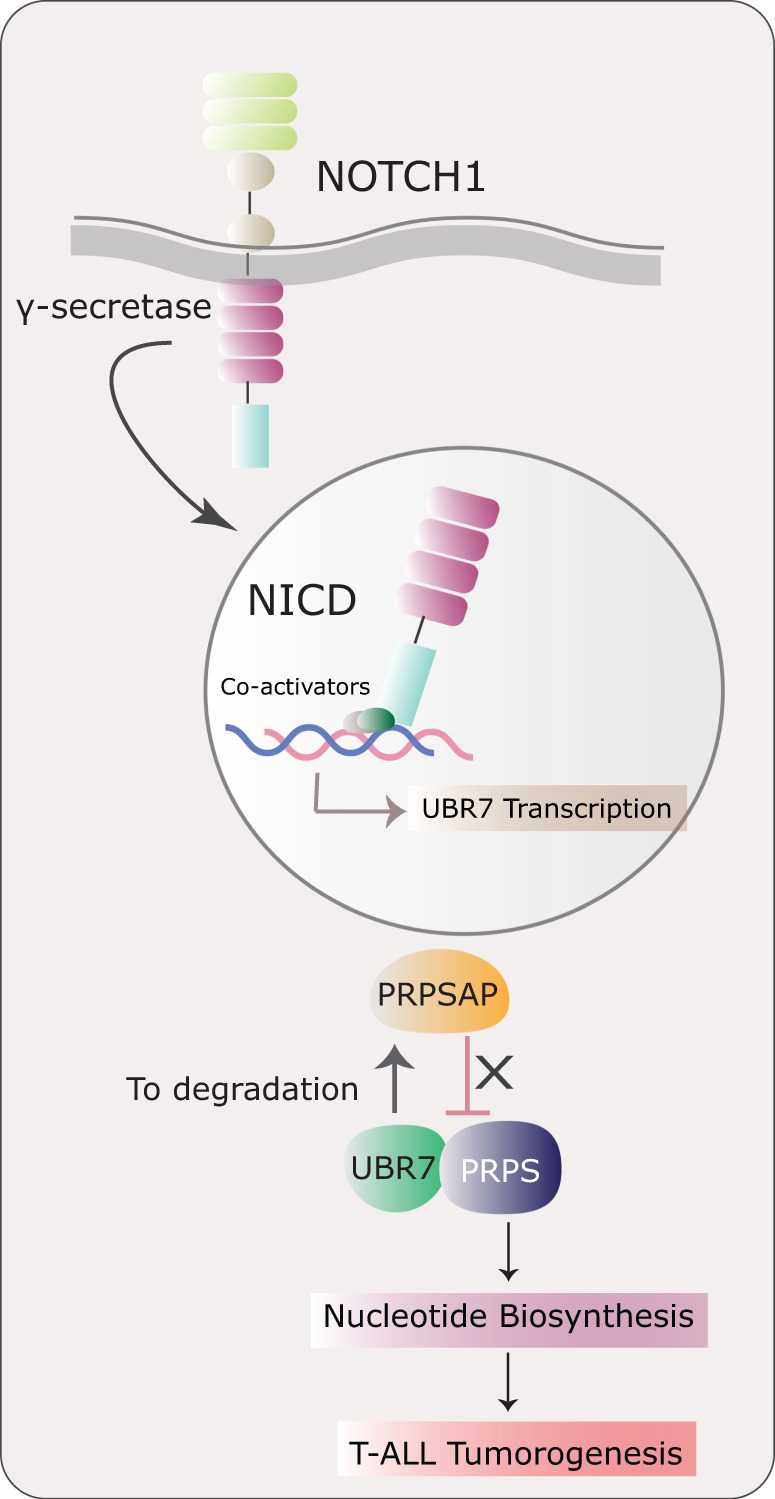
Nucleotide biosynthesis regulation by UBR7 in T-ALL. A model depicting the generation of NICD and its translocation to the nucleus from the proteolytic cleavage of NOTCH1 receptor by gamma secretase. NICD transcriptionally activates UBR7. UBR7 stabilizes the PRPS enzymes by promoting the degradation of PRPSAP. Stabilization of PRPS catalyzes the nucleotide biosynthesis and promotes T-ALL. In absence of UBR7, PRPSAP inhibits PRPS enzymes resulting in suppression of nucleotide biosynthesis and T-ALL progression. (NICD NOTCH intracellular domain; UBR7 ubiquitin protein ligase E3 component N-recognin 7; PRPS phosphoribosyl pyrophosphate synthetase; PRPSAP phosphoribosyl pyrophosphate synthetase associated protein; T-ALL T acute lymphoblastic leukemia).
